# Targeted Molecular Strategies for Genetic Neurodevelopmental Disorders: Emerging Lessons from Dravet Syndrome

**DOI:** 10.1177/10738584221088244

**Published:** 2022-04-13

**Authors:** Robert Lersch, Rawan Jannadi, Leonie Grosse, Matias Wagner, Marius Frederik Schneider, Celina von Stülpnagel, Florian Heinen, Heidrun Potschka, Ingo Borggraefe

**Affiliations:** 1 Department of Pediatrics, Division of Pediatric Neurology, Developmental Medicine and Social Pediatrics, University Hospital of Munich, Ludwig Maximilians University, Munich, Germany; 2 Institute of Human Genetics, University Hospital of Munich, Ludwig Maximilians University, Munich, Germany; 3 Institute of Human Genetics, Technical University of Munich, Munich, Germany; 4 Institute for Neurogenomics, Helmholtz Centre Munich, German Research Center for Health and Environment (GmbH), Munich, Germany; 5 Metabolic Biochemistry, Biomedical Center Munich, Medical Faculty, Ludwig Maximilians University, Munich, Germany; 6 International Max Planck Research School (IMPRS) for Molecular Life Sciences, Planegg-Martinsried, Germany; 7 Research Institute for Rehabilitation, Transition and Palliation, Paracelsus Medical Private University (PMU), Salzburg, Austria; 8 Institute of Pharmacology, Toxicology, and Pharmacy, Ludwig Maximilians University, Munich, Germany; 9 Comprehensive Epilepsy Center, University Hospital of Munich, Ludwig Maximilians University, Munich, Germany

**Keywords:** SCN1A, Dravet syndrome, precision medicine, epilepsy, therapy

## Abstract

Dravet syndrome is a severe developmental and epileptic encephalopathy mostly caused by heterozygous mutation of the *SCN1A* gene encoding the voltage-gated sodium channel α subunit Na_v_1.1. Multiple seizure types, cognitive deterioration, behavioral disturbances, ataxia, and sudden unexpected death associated with epilepsy are a hallmark of the disease. Recently approved antiseizure medications such as fenfluramine and cannabidiol have been shown to reduce seizure burden. However, patients with Dravet syndrome are still medically refractory in the majority of cases, and there is a high demand for new therapies aiming to improve behavioral and cognitive outcome. Drug-repurposing approaches for *SCN1A*-related Dravet syndrome are currently under investigation (i.e., lorcaserin, clemizole, and ataluren). New therapeutic concepts also arise from the field of precision medicine by upregulating functional *SCN1A* or by activating Na_v_1.1. These include antisense nucleotides directed against the nonproductive transcript of *SCN1A* with the poison exon 20N and against an inhibitory noncoding antisense RNA of *SCN1A*. Gene therapy approaches such as adeno-associated virus–based upregulation of *SCN1A* using a transcriptional activator (ETX101) or CRISPR/dCas technologies show promising results in preclinical studies. Although these new treatment concepts still need further clinical research, they offer great potential for precise and disease modifying treatment of Dravet syndrome.

## Introduction

**Figure 1.**
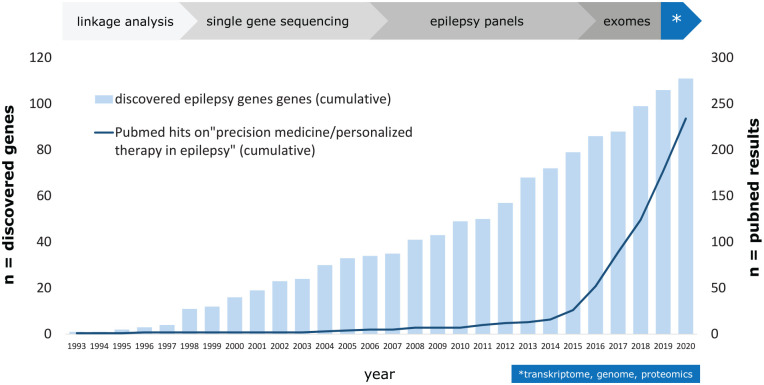
Evolution of precision medicine for epilepsy. With new methods of gene sequencing and their introduction into clinical application, many new epilepsy genes (light blue rectangles) were discovered over the past 20 years. Especially since 2015, there has been a significant increase in publications about precision medicine in the field of epilepsy treatment.

**Figure 2.**
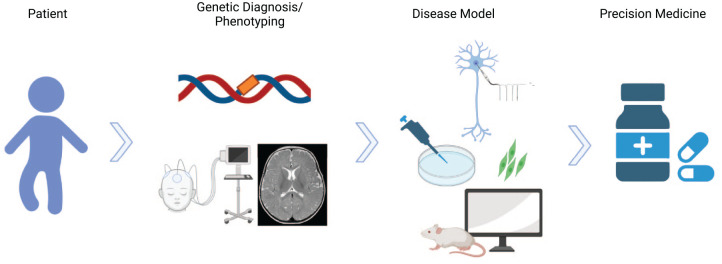
The concept of precision-based therapies in genetic epilepsy. A comprehensive knowledge of the genetic and epigenetic changes that lead to the development of the disease is needed. In a next step, preclinical in vitro and in vivo disease models are required for the development of drugs that can ultimately come into clinical use for specific genetic epilepsies.

*SCN1A*-related Dravet syndrome (DS) is an early onset developmental and epileptic encephalopathy (DEE) characterized by multiple seizure types, cognitive decline, behavioral disturbances, and ataxia. The incidence is estimated to be about 1:22,000 (Bayat and others 2015). The most common causes of DS are pathogenic heterozygous variants in *SCN1A*. This gene encodes the α subunit of the voltage-gated sodium channel type 1 Na_V_1.1 ion channel, which is predominantly expressed in the central nervous system on axons of fast-spiking GABAergic inhibitory interneurons (Ferreira Marques da Silva and others 2020). In some cases, variants in *SCN1A* cause milder phenotypes than DS. Recently, some new antiseizure medications (ASMs) were approved that revealed reduction of seizure frequency in a considerable number of patients with DS. Nevertheless, seizure freedom is only rarely achieved. Furthermore, significant comorbidities such as cognitive decline and behavioral disturbances may not improve in response to ASMs. Thus, there is an eminent need for new therapies directly targeting the genetic defect in *SCN1A*-related DS in order to improve the whole burden of all clinical symptoms. Disease-targeting precision medicine approaches are gaining relevance for the management of pediatric genetic epilepsies (Maljevic and others 2017; Syrbe 2021; Tacke and others 2017; von Stülpnagel and Kluger 2021). About 100 monogenic causes of epilepsy have been identified, and there are increasing reports on precision medicine approaches in these diseases (Fig. 1). The concept of precision medicine requires broad knowledge about disease models on a clinical, cellular, and molecular level in order to identify targeted therapeutic treatment options (Fig. 2). This review summarizes treatment options in *SCN1A*-related DS with special emphasis on evolving therapeutical approaches of personalized medicine. There are many works that particularly focus on current and future treatment options of DS (Brigo and others 2018; Johannessen Landmark and others 2021; Strzelczyk and Schubert-Bast 2020; Wirrell and Nabbout 2019). The goal of our study was to provide a comprehensive overview of DS, including its clinical features, genetics, pathophysiology, current treatment options, and future treatment approaches.

## Clinical Spectrum of *SCN1A*-Associated Epilepsies

### Dravet Syndrome (MIM: #607208)

*SCN1A*-related Dravet syndrome is an early onset developmental and epileptic encephalopathy characterized by multiple seizure types, cognitive decline, behavioral disturbances, and ataxia (Table 1). Development is normal within the first years of life and deteriorates subsequently. DS commonly manifests with prolonged febrile convulsions (typically hemiconvulsive) around eight months of age. In the further course, focal and generalized seizure types occur, including tonic-clonic, hemiclonic, atypical absence, myoclonic, and atonic seizures (EpilepsyDiagnosis.org n.d.). Electroencephalogram (EEG) is mostly normal at the time of seizure manifestation. During the disease course, generalized and focal epileptic discharges occur. In addition, analysis of the EEG reveals background slowing (Bureau and others 2011). Photoparoxysmal response may be observed in nearly 40% of children at the onset (Specchio and others 2012). Twenty-one percent of the patients with DS have epilepsy-related premature mortality, which is more than the percentage of deaths in other types of epilepsy (Genton and others 2011; Shmuely and others 2016). The majority of premature mortality is related to sudden unexpected death in epilepsy (SUDEP, up to half of the cases). Compared with other epilepsies, SUDEP in patients with DS occurs at younger age (73% before 11 years of age) (Sakauchi and others 2011). The second most common cause of death in DS, accounting for 32% of cases, is status epilepticus (Shmuely and others 2016).

**Table 1.** Disease Progression of Dravet Syndrome.^a^

Characteristic	Phase I	Phase II	Phase III
Synonym	Diagnostic stage	Deterioration stage	Plateau stage
Age	6 months to 1 year	1 to 5 years	>5 years
Seizure types	Prolonged febrile seizures/states (often hemiconvulsive)	Multiple seizure types: – Myoclonic seizures – Generalized convulsive seizures – Unilateral motoric seizures – Dialeptic seizures – Rarely: tonic seizures – Inclination to epileptic states	Convulsive seizures, predominantly at night
Interictal EEG	Background rhythm is normal Postictal unilateral slowing may occur after hemiconvulsive seizures Rarely interictal epileptic discharges Photoparoxysmal response in nearly 40% of children	Background rhythm in up to 50% of the cases reduced Photoparoxysmal response Generalized spike-wave and polyspike-wave complexes, focal spikes	Background rhythm remains usually reduced Photoparoxsmal response only rarely observed now Generalized spike-wave and polyspike-wave complexes occur less frequently
Cognition/behavior	Normal	Cognitive deterioration Onset of behavioral disorders	Cognitive degradation plateaus, behavior may improve slightly
Ataxia/gait	No abnormalities	Ataxia becomes increasingly apparent	Present; no further deterioration Crouch gait becomes evident
Other	Although it is called the “diagnostic” stage, the diagnosis of DS is occasionally not made yet		Significantly increased SUDEP risk (up to 10%)

DS = Dravet syndrome; EEG = electroencephalogram; SUDEP = sudden unexpected death in epilepsy.

a While DS is initially characterized by febrile convulsions, in stage II, various seizure types accompanied by symptoms such as cognitive decline, ataxia, and behavioral disturbances occur. Eventually, there is a plateau phase, in which, however, the cumulative risk for SUDEP increases. Photoparoxysmal response in the EEG may be observed in nearly 40% of children at the onset (Specchio and others 2012).

Most patients with DS experience cognitive deterioration within the first years of live (Guzzetta 2011). It is a matter of debate which factors have the most significant impact on patients’ cognitive decline. Both studies from DS patients and animal data suggest a minor effect of seizures on cognition in DS (Bender and others 2013; Nabbout and others 2013). More likely, sodium channel dysfunction itself appears to be the most relevant factor causing cognitive disturbances. In addition, significant adverse effects on cognitive performance and behavior have been shown for many ASMs and commonly reveal cumulative effects when drugs are combined (which is the case in DS in most cases) (Andrew and others 2012; Besag and others 2016).

## Allelic Diseases Associated with Variants in *SCN1A* Other Than DS

Besides diseases with MIM designation, one has to acknowledge that some of the following terms without MIM designation such as “PEFS+” and “focal epilepsy” may appear within the literature but are not be generally accepted.

### GEFS+ (MIM: #60440)

The name of generalized epilepsy with febrile seizures plus (GEFS+) has been recently changed to “genetic epilepsy with febrile seizures plus” in order to include focal seizures that may occur in this group (Camfield and Camfield 2015). GEFS+ is associated with heterogeneous epilepsy phenotypes (Riva and others 2021). Patients with GEFS+ may present with febrile seizures or with afebrile generalized tonic-clonic seizures in childhood with remission in adolescence. The cognitive and behavioral prognosis is favorable.

### *SCN1A* DEE Other Than DS (MIM: #619317)

Patients with *SCN1A* DEE have an earlier age of onset, a profound developmental impairment, and severe hyperkinetic movement disorders compared to patients with DS (Sadleir and others 2017).

### Familiar Hemiplegic Migraine (MIM: #609634)

*SCN1A* mutations may also cause familiar hemiplegic migraine (FHM). Migraine attacks usually comprise aura (i.e., unilateral sensory loss) and further focal neurological signs such as hemiplegia, aphasia, and visual disturbances (Dichgans and others 2005; Scheffer and Nabbout 2019).

### Partial Epilepsy with Febrile Seizures Plus

Patients with partial epilepsy with febrile seizures-plus (PEFS+) present with febrile seizures and late-onset epilepsy. They may develop exclusively focal seizures with or without fever in the first years of life (Camfield and Camfield 2015). For the reasons mentioned above, PEFS+ is now included within the GEFS+ group.

### Focal Epilepsy

A distinct phenotype of focal epilepsy was recently suggested in individuals in whom focal-onset seizures were the only form of seizures. These patients can be distinguished from patients with PEFS+ as they develop pharmacoresistant focal epilepsies, which is uncommon in PEFS+ (Laur and others 2021).

### Other

In rare cases, heterozygous *SCN1A* mutations might cause myoclonic-atonic epilepsy or epilepsy of infancy with migrating seizures (Scheffer and Nabbout 2019).

For a more detailed review of *SCN1A*-associated phenotypes, the interested reader is referred to Scheffer and Nabbout (2019).

## Genetics of *SCN1A*-Associated Disorders

### The *SCN1A* Gene

The *SCN1A* gene has 26 exons encoding the α subunit of the neuron voltage-gated sodium channel type 1 (Na_v_1.1). *SCN1A* is already expressed in embryonic stages, and expression increases particularly postnatally and then gradually until adulthood (Catterall and others 2010). On a cellular level, the *SCN1A* gene is predominantly expressed in dendrites, in cell bodies, and at the axon hillock of fast-spiking inhibitory GABAergic interneurons within the central nervous system (Escayg and Goldin 2010; Ferreira Marques da Silva and others 2020; Meisler and Kearney 2005). On a regional level, *SCN1A* is mainly expressed within the frontal cortex forebrain, hippocampus, and cerebellum (Uhlen and others 2015; Wang and others 2011). This regional distribution is thought to contribute to the clinical symptoms such as cognitive and behavioral disturbances, seizures, and ataxia, respectively.

### Inheritance and Types of Variants

The inheritance pattern of all currently known *SCN1A*-associated conditions is autosomal dominant. In approximately 70% of *SCN1A*-related DS, mutations arise de novo (De Jonghe 2011). The *SCN1A* mutations in DS are most commonly truncating or missense mutations. Truncating mutations, which cause protein function loss and a severe phenotype, account for half of the DS-causing mutations. Missense mutations account for the other half and often result in reduced protein function, leading to a variety of phenotypes ranging from severe to mild (De Jonghe 2011). The truncating mutations lead to a loss of function due to frameshift, nonsense, insertion/deletion, rearrangement, and splice site mutations. Either way, loss of function of one allele is the most likely consequence of most cases of *SCN1A*-related DS.

Previous studies in mice and in cell lines indicate that Na_V_1.1 truncating mutations have no dominant negative effect (Bechi and others 2012; Yu and others 2006). These findings underline treatment options aiming to increase the expression of the healthy allele in DS. Nevertheless, cases of a gain-of-function mutation with an even more severe clinical phenotype than DS have been described recently (Berecki and others 2019). Consequently, latter cases will not qualify for treatment approaches that enhance gene expression of the intact allele.

### Genotype-Phenotype Correlations

Since many different types of mutations of *SCN1A* cause distinct clinical phenotypes, several studies have been conducted to investigate putative genotype-phenotype correlations. Patients with GEFS+ and patients with febrile seizures only are more likely to have a missense mutation than a truncating mutation (Zuberi and others 2011). In DS, both missense and truncating mutations are detectable. When missense mutations are associated with a DS phenotype, they are most likely located in functionally highly relevant locations (i.e., the pore-forming region, likely causing a loss of function) (Catterall and others 2010; Meisler and Kearney 2005). Nonsense and frameshift mutations lead by truncation to a haploinsufficiency of Na_v_1.1 (Meisler and Kearney 2005). Hence, it can be summarized that the clinical significance of specific *SCN1A* mutations depends on the type of mutation and its location within the gene (Zuberi and others 2011). However, some patients show a milder phenotype and disease progression than others carrying the same mutation, suggesting the additional presence of genetic modifiers (Depienne and others 2009, 2010; Guerrini and others 2010; Miller and others 2014; Nabbout and others 2003; Osaka and others 2007; Riva and others 2021; Yu and others 2010). The exact functional implications of these are nonetheless still largely unknown.

## Neuroimaging in DS

Some patients with DS may have structural brain abnormalities. These comprise general or focal brain atrophy, cortical dysplasia, and hippocampal sclerosis (Barba and others 2014; Skjei and others 2015; Tiefes and others 2019). Hippocampal sclerosis might be attributed to repeated febrile status epilepticus in DS patients. The occurrence of focal structural brain abnormalities in DS prompts the question of whether these patients are eligible candidates for resective epilepsy surgery. However, these approaches have been tried for DS patients with dysplasia and did not reveal a worthwhile improved seizure outcome in most patients. A recent survey of eight patients suggested that only patients with a milder phenotype of *SCN1A*-related epilepsy than DS may profit from resective epilepsy surgery in some circumstances (Vezyroglou and others 2020).

## Molecular Neurophysiology of *SCN1A*-Related DS

### The Na_v_1.1 Channel and Interneuron Model

**Figure 3.**
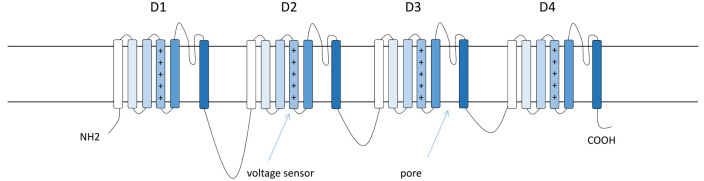
Structure of the α type I sodium channel (Na_v_1.1). Na_v_1.1 is composed of a 260-kDa α subunit and auxiliary β subunits (β1–β4) of 33 to 36 kDa. The α subunit consists of four internally homologous domains (D1–D4). Each domain contains six α-helical transmembrane segments. Between the fifth and the sixth segments, there is an ion-conducting pore loop (Catterall and others 2010).

**Figure 4.**
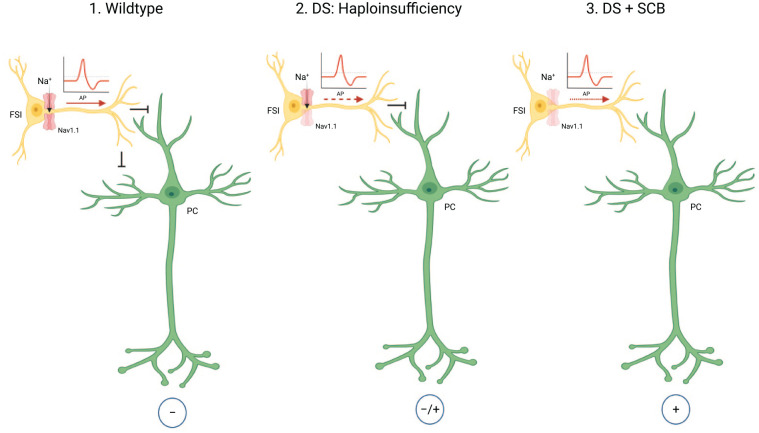
Interneuron model in Dravet syndrome (DS). Na_v_1.1 is mainly expressed on axon hillocks of fast-spiking inhibitory interneurons, and heterozygous *SCN1A* mutations will most likely lead to a loss of excitability of GABAergic inhibitory interneurons, resulting in hyperexcitability of downstream pyramidal neurons. Sodium channel blocker (SCB) can cause further seizure deterioration in DS patients due to the inhibition of the remaining functionally intact channels (de Lange and others 2018; Oakley and others 2011; Yu and others 2006). AP = action potential; FSI = fast-spiking GABAergic interneuron; PC = pyramidal cell. Adapted from Jensen and others (2014). Figures were created using BioRender©.

The *SCN1A* gene encodes the α type I sodium channel (Na_v_1.1). It is predominantly located at axon hillocks of fast-spiking interneurons (Figs. 3 and 4). The primary structure of a voltage-gated sodium channel is composed of a 260-kDa α subunit and auxiliary β subunits (β1–β4) of 33 to 36 kDa. The α subunit consists of the voltage sensors and four internally homologous domains that form the ion-conducting pore (Catterall and others 2010). Each domain contains six potential α-helical transmembrane segments (S1–S6). Between S5 and S6, there is an ion-conducting pore loop (Fig. 3) (Catterall and others 2010). Yu and colleagues (2006) showed that the sodium current density in inhibitory interneurons was lower in *Scn1a*^–/–^ and *Scn1a*^+/–^ mice compared to wild-type (WT) mice, while voltage-dependent activation and inactivation was not altered.

Given the concept of haploinsufficiency, heterozygous *SCN1A* mutations will most likely lead to a loss of excitability of GABAergic inhibitory interneurons, resulting in hyperexcitability of downstream dentate granules and pyramidal neurons (Oakley and others 2011; Yu and others 2006). A wide variety of antiseizure medications that act as modulators of voltage-gated sodium channels (sodium channel blocks [SCBs]) have been shown to inhibit Na_v_1.1 function. Consequently, these drugs commonly cause seizure deterioration in DS patients due to the inhibition of the remaining functionally intact channels (Fig. 4). Even more, DS patients with SCB treatment over a considerable time period exhibit a worse cognitive outcome compared to DS patients who did not receive SCBs (de Lange and others 2018).

## Current Therapeutic Options in DS

In most cases, once the diagnosis of DS is established, valproic acid (VPA) with or without clobazam (CLB) is usually introduced (Cardenal-Munoz and others 2022). A combination of CLB, valproate, and stiripentol has a considerable effect on reducing convulsive and prolonged seizures, although cumulative side effects are common (Cross and others 2019). Others prefer topiramate as second-line treatment. However, cognitive deterioration and weight loss might limit the use of topiramate (Eddy and others 2011). Eventually, the introduction of new drugs such as fenfluramine and cannabidiol or the reconsideration of bromide in some countries may most likely alter treatment guidelines for DS very soon (see below) (Strzelczyk and Schubert-Bast 2020).

### Fenfluramine

Fenfluramine was originally approved as a weight loss drug. However, the drug was withdrawn from the market in the late 1990s due to the occurrence of heart valve disease and pulmonary arterial hypertension in some patients (Abenhaim and others 1996; Connolly and others 1997). Small open-label studies and case reports described the use of fenfluramine for photosensitive epilepsy and self-induced seizures and suggested an anticonvulsant activity of the drug (Aicardi and Gastaut 1985; Schoonjans and others 2015). The US Food and Drug Administration (FDA) and the European Commission authorized fenfluramine in 2020 as an add-on therapy to other ASMs for treatment of seizures associated with DS in individuals aged two years and older. In two multinational randomized placebo-controlled phase 3 studies and in an open-label extension study, fenfluramine significantly reduced seizure frequency in DS patients (responder rate of 54% to 72.9%) compared to placebo (Lagae and others 2019; Nabbout and others 2020; Sullivan and others 2020). The responder rate (50% or more seizure reduction) of convulsive seizures was sustained in most patients (>70%) after a period of 21 months (Sullivan and others 2020). Fenfluramine was usually well tolerated, with no negative effects on the cardiovascular system. Fenfluramine and its active metabolite norfenfluramine have a primarily serotonergic mechanism of action, with 5-HT release and more selective activation of 5-HT1D and 5-HT2C receptors (Schoonjans and Ceulemans 2021). Moreover, an interaction of fenfluramine with Sigma1 might be of additional relevance (Martin and others 2021). Besides significant seizure reduction, fenfluramine might even improve intellectual skills (Bishop and others 2021).

### Cannabidiol

Two compounds present in the resin of the marijuana plant are tetrahydrocannabinol (THC) and cannabidiol (Iannone and others 2021). THC is responsible for marijuana’s psychoactive properties. Cannabidiol (CBD), which has a very similar molecular structure to THC and makes up about 40% of cannabis extract, has no psychoactive properties. CBD has been shown to reduce psychotic symptoms, anxiety, inflammation, nausea, and seizures (Iannone and others 2021). The antiseizure effect of CBD is most likely caused by reduction of neuronal excitability by inhibiting GPR55 and TRPV1 receptors and modulating adenosine signaling (Gray and Whalley 2020; Sylantyev and others 2013). In a preclinical DS model, CBD-treated mice showed a reduction in seizure frequency and autistic-like social behavior (Kaplan and others 2017). In this study, use of a GPR55 receptor antagonist mimicked these effects, suggesting that CBD’s mode of action is mediated through this receptor. Most likely by inhibition of the cytochrome P450 subtype 2C19, CBD leads to increased levels of the active metabolite of CLB (N-desmethylclobazam) (Devinsky and others 2018). Therefore, caution should be exercised when CBD is combined with CLB and CLB levels and its metabolites should be regularly monitored. It has previously been suggested that the anticonvulsant efficacy of CBD in refractory epilepsy is merely due to elevated CLB plasma levels (Anderson and others 2019; Devinsky and others 2017). However, a recent meta-analysis showed that it is much more likely that CBD has an intrinsic efficacy independent from the CLB addition (Lattanzi and others 2020b). Adjunctive CBD resulted in a greater reduction in convulsive seizure frequency than placebo and was associated with a higher rate of adverse effects in DS (Lattanzi and others 2020a). In a double-blind, placebo-controlled study including 120 children and young adults, CBD as an adjunctive medication was able to significantly reduce the frequency of seizures per month from 12.4 to 5.9 compared to a decrease from 14.9 to 14.1 within the placebo group. The proportion of responder (patients who reached at least 50% seizure reduction of convulsive seizures) was higher within the CBD compared to the placebo group, although not reaching statistical significance (43% vs. 27%, respectively) (Devinsky and others 2017). Longer follow-up studies have shown a significant reduction in seizure frequency and mostly mild to moderate side effects such as diarrhea, fever, decreased appetite, and somnolence (Devinsky and others 2019).

### Stiripentol

Stiripentol was approved in the European Union (EU) in 2007. The mechanism of action is most likely through enhancement of inhibitory neurotransmission and an increase of extracellular GABA levels. Stiripentol also induces and inhibits different cytochrome P450 (CYP) enzymes. Sometimes, the dose of CLB must be reduced due to increased plasma concentrations. Stiripentol is often used in combination with valproic acid and clobazam. A trial demonstrated a significant reduction in seizure frequency when combined with VPA and CLB (Chiron and others 2000).

### Bromide

Bromide is a helpful agent in the treatment of epilepsy, particularly in patients who are refractory to conventional ASMs. At least a 50% reduction in seizure frequency has been documented in up to 77% of patients, when combined with other treatments (Lotte and others 2012). Bromide is a putative candidate for drug combinations, because it has no pharmacokinetic interactions with other ASMs (Ryan and Baumann 1999). According to a retrospective study of 99 Japanese patients with DS, 41.7% of those who received bromide were protected against the development of status epilepticus (Tanabe and others 2008). Regular monitoring of bromide serum concentrations along with nutritional monitoring of sodium chloride intake is essential to avoid side effects. The use of bromide might be limited by its cognitive and dermatologic side effects (Kodama and others 2019). Although the specific mechanism of action of bromide is unknown, the most plausible mode of action is the stabilization of excitable membranes through hyperpolarization of neurons (Ryan and Baumann 1999). However, it has to be acknowledged that there are no randomized controlled trials on the effect of bromide on DS yet.

### Rufinamide

Rufinamide is an ASM that has been shown to be effective as an adjunctive therapy in the treatment of seizures associated with Lennox-Gastaut syndrome (Glauser and others 2008; Kluger and others 2010; Wheless and Vazquez 2010). The mode of action is most likely due to the prolongation of the inactivation phase of voltage-gated sodium channels (Striano and others 2018). In a retrospective European multicenter study, the response rate in DS patients was 20% at 6 months and 5% at 34 months, and 30% discontinued treatment due to aggravation of seizures (Mueller and others 2011).

### Levetiracetam

Levetiracetam is an ASM that is widely used in children and has a broad spectrum of activity. Its mode of action includes, among other things, the binding of the synaptic vesicle protein SV2A (Lynch and others 2004). A prospective open-label add-on trial in patients with DS showed a response rate of 64.2% for tonic-clonic, 60% for myoclonic, 60% for focal, and 44.4% for absence seizures with a reduction of more than 50% (Striano and others 2007). A more recent retrospective study showed a response rate of 30% with a reduction of seizures of more than 50% in DS patients (Dressler and others 2015).

### Ketogenic Diet

For therapy refractory epilepsies, a ketogenic diet (KD) may be considered. KD aims to induce a ketogenic state in the body through a high-fat, low-carb, and moderate-protein diet (Wells and others 2020). KD has been shown to have an anticonvulsant effect in *Scn1a* mutant mice by increasing fluorothyl-induced seizure thresholds (Dutton and others 2011). A reduction in seizure frequency was observed in more than half of the patients with DS in several studies (Caraballo 2011; Caraballo and others 2005; Dressler and others 2015; Laux and Blackford 2013; Nabbout and others 2011; Tian and others 2019; Yan and others 2018). The mode of action may be due to neuroprotective effects as well as changes in neurotransmitter levels as a result of the ketogenic state (Youngson and others 2017). Another mode of action could be the modulation of the microbiome through the ketogenic diet (Miljanovic and Potschka 2021). Although no serious side effects have been described, noncompliance appears to play an important role, particularly in older children (Dressler and others 2015).

### Vagus Nerve Stimulation

Vagus nerve stimulation (VNS) was approved in the 1990s for the adjunctive treatment of drug-resistant epilepsy and works by stimulating the left cervical vagus nerve through an implanted electrode connected to a pulse generator (Dibue-Adjei and others 2017). A meta-analysis of 13 studies reported a reduction in seizure frequency of more than 50% in about half of the included DS patients (Dibue-Adjei and others 2017). VNS therapy also showed long-term improvement in seizure control. The most common reported side effect was hoarseness (Youn and others 2021).

### Rescue Treatment for Prolonged Seizures

Patients with DS often develop prolonged convulsive seizures that require emergency treatments. These are often patient specific. In general, seizures lasting longer than five minutes should be interrupted, although depending on the patient, an earlier administration of rescue medication may be advisable (Cross and others 2019). Preclinically, depending on the country, midazolam or diazepam is administered buccally or rectally. Midazolam or clonazepam intravenously might be considered next. If the seizure still persists, valproate, levetiracetam, or phenytoin may be administered. Finally, anesthesia is induced with, for example, ketamine or phenobarbital (Cross and others 2019).

## Future Therapeutic Strategies

There are several new therapeutic concepts to treat *SCN1A*-related DS. One promising approach is drug repurposing. Drug repurposing aims to identify drugs that are already approved for other medical indications. The approach offers several advantages. The timeframe until approval is often significantly shorter and the risk of failure is lower, because the medication has already been shown to be safe in preclinical and clinical studies. Also, the costs are often lower (Ashburn and Thor 2004; Pushpakom and others 2019). The most important therapy concepts from the field of drug repurposing and precision medicine are reviewed below. For the majority of these approaches, clinical data are not yet available. Nevertheless, the mode of action and the current state of investigation are summarized. This list does not implicate a fully comprehensive overview of all currently investigated agents, and readers might be encouraged to seek any new updates (e.g., on https://clinicaltrials.gov/).

## Drug Repurposing

### Lorcaserin (EPX-200)

Lorcaserin was approved by the FDA in 2012 for treatment of obesity. The effect is based on selective activation of 5-HT2C serotonin receptors (Sharma and others 2020). Selective modulators of serotonin signaling have been shown to successfully reduce seizure activity in zebrafish larvae with *SCN1A* mutations and patients with DS (Griffin and others 2017). Lorcaserin is currently in a phase 3 study for treatment of DS (NCT04572243).

### Clemizole (EPX-100)

Clemizole is a potent H1 receptor antagonist, which was commonly used for treatment of allergic diseases (Jacques and Fuchs 1960; Zierz and Greither 1952) and was shown to suppress hepatitis C virus replication (Einav and others 2008). The reduction of electrographic seizures by this compound was first found in a drug screen in zebrafish with a mutation of the scn1Lab sodium channel, which displays a strong homology with human *SCN1A* (Baraban and others 2013). H1 receptor antagonists are usually contraindicated in children with epilepsy (Miyata and others 2011). However, the mode of action of clemizole in DS seems to be due to inhibition of HTR2A and HTR2B receptors (Griffin and others 2017). The efficacy of EPX-100 for treatment of DS is currently investigated in a phase 2 study (NCT04462770).

### Huperzine Analog (BIS-001)

Huperzine was originally isolated from *Huperzia serrata* and is a potent inhibitor of acetylcholinesterase (Ma and others 2007). This compound was proved to be effective and safe in several neurological disorders, including Alzheimer disease, schizophrenia, and vascular dementia (Wang and others 2009; Xu and others 2012; Zhang and others 2007). Huperzine was shown to reduce seizures in *Scn1a* mutant mice (Wong and others 2016) but not in a zebrafish model of DS (Dinday and Baraban 2015). Its anticonvulsant effect may be based on neuroprotective and anti-inflammatory processes by inhibition of acetylcholinesterase and antagonization of the NMDA receptor (Wong and others 2016). The effect of BIS-001 for treatment of epilepsy was investigated in a phase 1 trial (NCT03156439).

### Ataluren

Ataluren targets genetic disorders by interacting with translation and preventing premature termination caused by early stop codons (Welch and others 2007). It is approved in the EU for treatment of nonsense mutation-mediated Duchenne muscular dystrophy (Berger and others 2020), and its effectiveness in patients with cystic fibrosis is currently being assessed (Konstan and others 2020). The efficacy of ataluren in DS resulting from a nonsense mutation was investigated in a phase 2 trial (NCT02758626). However, available clinical data from a small patient group argue against a relevant clinical efficacy (Devinsky and others 2021).

### Verapamil

Verapamil is a voltage-gated calcium channel blocker often used to treat hypertension and certain kinds of cardiac arrhythmia (Strzelczyk and Schubert-Bast 2020). It showed some effect as an add-on drug for treatment of drug-resistant epilepsies in children (Nicita and others 2014). The mode of action is most likely based on the increased uptake of ASMs in the brain by inhibiting the multidrug transporter P-glycoprotein (Pgp) and by hindering an increased influx of calcium into the neurons, which presumably leads to membrane hyperexcitability (Nicita and others 2014). However, its use as a broad drug efflux transporter inhibitor may be subject to adverse effects (Nicita and others 2014). The safety and the effect on seizure reduction in DS is being investigated in a phase 2 trial (NCT01607073).

## The Urgent Need for Disease-Modifying Drugs

Seizures in DS are resistant to therapy. New medications for the treatment of DS, such as cannabidiol and fenfluramine, can significantly reduce seizure frequencies in a relevant proportion of patients. For fenfluramine, studies even suggest an improvement in intellectual skills (Bishop and others 2021). Nevertheless, despite advancements in the management of the disease, most patients continue to experience seizures. In addition, there is still a pressing need to develop new therapies that not only treat seizures but also improve behavioral and cognitive symptoms. Recurrent seizure activity in DS does not explain all behavioral problems and cognitive deterioration alone. The *SCN1A* mutation itself seems to play a significant role in line with the general concept of DEEs. A recent study revealed that DS patients with *SCN1A* mutation showed a more severe psychomotor delay than those without mutation (Nabbout and others 2013). Furthermore, typical epilepsy-related parameters such as frequency of convulsive status were not related to a more severe cognitive phenotype. As a necessary consequence, new treatment options are required to overcome all symptoms caused by *SCN1A* mutation.

## Precision Medicine

### Na_V_1.1 Activating Drugs

Na_V_1.1 activators may have great potential in the treatment of DS by improving the function of fast-spiking GABAergic interneurons (Jensen and others 2014). One such drug is AA43279; it has already been shown to be effective in vitro and in vivo with moderate selectivity for Na_V_1.1 (Frederiksen and others 2017). Newer components such as the spider venom peptides Hm1a and Hm1b, which are more stable in serum and cerebrospinal fluid, show a higher selectivity for Na_V_1.1 in preclinical studies (Chow and others 2020; Richards and others 2018).

### Soticlestat (TAK-935/OV935)

Soticlestat is a novel inhibitor of the brain-specific cholesterol 24-hydroxylase and reduces the conversion of cholesterol into 4S-hydroxycholesterol (24HC) (Hawkins and others 2021; Nishi and others 2020). In mice, this compound decreased neural excitability by significantly reducing the amount of 24HC in the brain (Hawkins and others 2021; Nishi and others 2020). In *Scn1a*^+/–^ DS mice, soticlestat reduced seizures and SUDEP, as well as protected against hyperthermia-induced seizures (Hawkins and others 2021). The efficacy as an add-on therapy in children and young adults with DS will be evaluated in a phase 3 trial (NCT04940624). First clinical efficacy data from a phase 1b/2a study in participants with DEEs have been reported, demonstrating a relevant reduction in seizure frequency by 36.4% in the maintenance phase (Halford and others 2021).

### Antisense Oligonucleotides

Antisense oligonucleotides (ASOs) consist of a single-stranded base sequence that can target specific RNA or DNA molecules, thereby modulating the expression of mutant proteins encoded by the targeted transcript (Evers and others 2015).

#### STK-001/targeted augmentation of nuclear gene output

**Figure 5.**
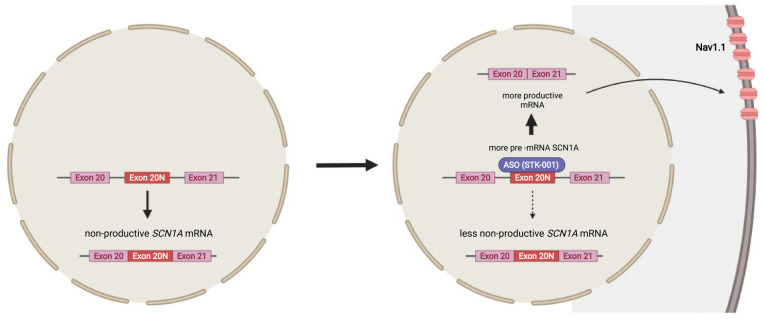
Targeting non-productive *SCN1A* transcript with inclusion of poison exon 20N with an antisense oligonucleotide (ASO). A naturally occurring alternative splicing event results in a nonproductive mRNA transcript of *SCN1A* with inclusion of poison exon 20N (Carvill and others 2018; Voskobiynyk and others 2021). An ASO (STK-001) directed against exon 20N promotes exon skipping, enhances the amount of productive mRNA, and increases the expression of sodium channel Na_v_1.1 protein expression (Hill and Meisler 2021; Isom and Knupp 2021). Figures were created using BioRender©.

Some rare disease-associated *SCN1A* mutations reside in intron 20 of the gene, leading to Na_V_1.1 loss of function by inclusion of a poison exon 20N by alternative splicing (Carvill and others 2018; Voskobiynyk and others 2021) (Fig. 5). This association has provided knowledge about the putative role of the poison exon 20N in other DS cases, as it occurs naturally as a nonproductive splicing event (Helbig and Goldberg 2021; Voskobiynyk and others 2021). Although it is believed that expression of poison exon accounts for only 1% of all *SCN1A* transcripts, this amount might be particularly higher at younger ages (Helbig and Goldberg 2021; Voskobiynyk and others 2021). The target of the investigational product STK-001 is the alternative nonproductive splice product by binding the poison exon 20N (Fig. 5) (Carvill and others 2018; Hill and Meisler 2021). This leads to the upregulation of the wild-type transcript. Thus, STK-001 has the potential to be the first disease-modifying medication to target DSs genetic etiology (Han and others 2020) and is currently being investigated in a phase 1 clinical trial (NCT04740476) (Laux 2021). The technology of targeted augmentation of nuclear gene output (TANGO) was used to design this drug (Han and others 2020). In a DS mouse model, STK-001 reduced the occurrence of seizures and SUDEP and revealed both prolonged survival and rescued interneuron excitability (Han and others 2020; Wengert and others 2022).

#### IncRNA *SCN1A*-dsAS as a target for antisense oligonucleotides

**Figure 6.**
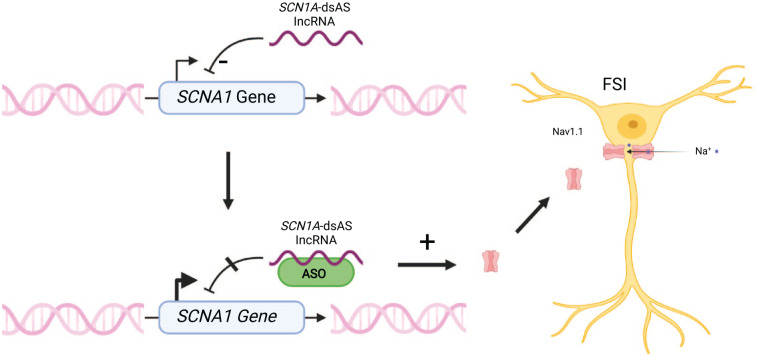
Precision medicine in Dravet syndrome by targeting *SCN1A-dsAS* with an antisense oligonucleotide (ASO). *SCN1A-dsAS* is an antisense long noncoding RNA negatively correlated with the expression of *SCN1A* in human brain tissue. It seems to inhibit transcription of *SCN1A*. An ASO directed against this long noncoding RNA leads to enhanced expression of wild-type *SCN1A* and increases the expression of Na_v_1.1 on fast-spiking inhibitory interneurons (Hsiao and others 2016). FSI = fast-spiking GABAergic interneuron. Figures were created using BioRender©.

Long noncoding RNAs (lncRNAs) are RNA transcripts that do not code for proteins and are by definition longer than 200 nucleotides (Quinn and Chang 2016). They have a key role in gene regulation. Antisense transcripts are a subtype of these lncRNAs, which are transcribed from the opposite strand of a sense transcript of a protein coding gene (Pelechano and Steinmetz 2013). Two of these antisense RNAs are located on the opposite strand of the *SCN1A* gene, and enhanced expression of *SCN1A* was recently shown in human fibroblasts of DS patients, in mice, and in monkeys by using antisense oligonucleotides directed against the lncRNA *SCN1A-*dsAS (downstream lncRNA) (Hsiao and others 2016). Improvement in seizure control in mice and a normalization of the neuronal firing of inhibitory interneurons were observed (Hsiao and others 2016). Both downstream and upstream *SCN1A* antisense RNAs are widely expressed within pediatric brain specimens, and lncRNA *SCN1A-*dsAS expression was shown to be negatively correlated with the expression of *SCN1A*, suggesting a role in inhibiting *SCN1A* expression (Borggraefe 2021). Thus, ASOs targeted against *SCN1A*-related lncRNAs are thought to increase *SCN1A* wild-type expression (Fig. 6). An antisense oligonucleotide against regulatory *SCN1A* RNA labeled “CO-3527” underwent preclinical evaluation and is stated by the investigating biotechnology company to be evaluated clinically for FDA application soon (Giagtzoglou 2021).

#### *SCN8A* ASO

*SCN8A* is a gene encoding for the sodium channel Na_v_1.6. Gain-of-function mutations in this channel can lead to neuronal hyperexcitability and epilepsy (Meisler 2019). Reduced expression of Na_v_1.6 might lead to reduced seizure susceptibility in *SCN1A*-linked DS, and therefore Na_v_1.6 might play a role as a genetic modifier. Recently, *SCN8A* ASO was developed, and it was shown that reduction of *SCN8A* expression led to reduced seizures and SUDEP in a DS mouse model (Isom and Knupp 2021; Martin and others 2007; Meisler 2019).

### Gene Therapy and Further Strategies

The goal of gene therapy is the genetic engineering of a cell by regulating, repairing, replacing, adding, or deleting genetic material in order to achieve a therapeutic effect (Kaji and Leiden 2001; Wirth and others 2013). For this purpose, especially viral vectors such as lentivirus or adeno-associated virus (AAV) were used in other genetic diseases (High and Roncarolo 2019). In *SCN1A*-related DS, the goal is to compensate for the haploinsufficiency of *SCN1A*. However, *SCN1A* is too large to be packaged into currently available viral systems, including AAV, making direct expression of the gene from a viral vector difficult (Yamagata and others 2020).

#### ETX 101

The advantage of AAV-based vector delivery systems concerning safety is that the vector is not able to replicate and does not integrate into the genome (High and Roncarolo 2019). A developer of precision gene therapies plans to treat the first patient in a clinical trial in 2022 with ETX 101, an AAV serotype 9 vector-based agent (Belle 2020). It delivers a *SCN1A*-specific transcription factor (eTF^SCN1A^) together with a GABAergic regulatory element for enhancement of *SCN1A* expression specifically in affected inhibitory neurons (Juando-Prats and others 2021). It decreased seizure frequency and SUDEP in a DS mouse model and was already tested in nonhuman primates (Juando-Prats and others 2021).

#### AAV-Navβ1

The α subunit of Na_v_1.1 cannot be overexpressed by AAV-based vectors, because the size of the coding region exceeds the capacity of AAV delivery (Niibori and others 2020). In a mouse model of DS, scientists tried to compensate for the reduced expression of Na_v_1.1 channels by overexpressing the significantly smaller β1 subunit of the sodium channel with the help of a *Gad1* promoter (Niibori and others 2020). The β1 subunit most likely modulates gating and expression of the α subunit on the plasma membrane (Calhoun and Isom 2014). Mice treated with an intrathecal injection of AAV-Navβ1 showed reduced spontaneous seizures and a longer survival compared to untreated mice. Interestingly, the effect was more pronounced in female mice (Isom and Knupp 2021; Niibori and others 2020).

#### CRIPSR/dCas9

**Figure 7.**
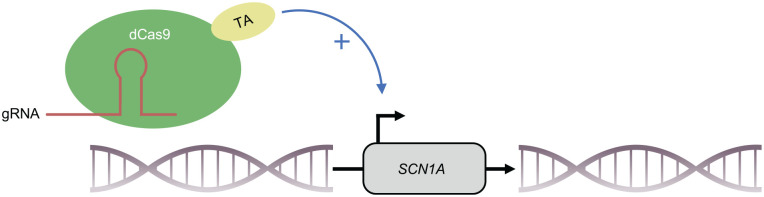
Activation of *SCN1A* transcription with CRISPRa. With a guide RNA complementary to the promoter sequence of *SCN1A*, a catalytically inactive Cas9 (dCas9) fused to a transcriptional activator can be brought to the *SCN1A* promoter locus and leads to enhanced transcription of the gene. With this system, researchers were able to increase the expression of *SCN1A* wild-type mRNA in the brain of DS mice (Colasante and others 2020; Yamagata and others 2020). gRNA = guide RNA; TA = transcriptional activator.

Cas9 can be brought to any specific locus of the genome with a protospacer-adjacent motif (PAM) with the help of a guide RNA (gRNA) complementary to the target sequence (Cong and others 2013; Jinek and others 2012; Mali and others 2013). Transcriptional activator domains fused to a nuclease-deactivated Cas9 protein (dCas9) can be brought into the promoter area of specific genes in order to activate their transcription (Chavez and others 2015; Cheng and others 2013; Gilbert and others 2013; Konermann and others 2015; Maeder and others 2013; Perez-Pinera and others 2013; Tanenbaum and others 2014). Recently, activation of *Scn1a* in vivo, using VP64 as a transcriptional activator fused to a dCas9, has been demonstrated (Colasante and others 2020). Furthermore, this approach was capable of significantly lowering the threshold temperature and the severity of seizures in *Scn1a*^+/–^ mutant mice. Using newer generations of transactivators (dCas9-VPR), almost normal *SCN1A* mRNA expression in inhibitory neurons was achieved in vivo (Yamagata and others 2020). This resulted in a lower threshold of hyperthermia-induced seizures, as well as a later onset and shorter duration of seizures in DS mice. In both studies, AAV was used as a vector (Colasante and others 2020; Yamagata and others 2020). Even if the studies show some limitations, the findings suggest that it might be worthwhile to further assess CRISPR/dCas9-based activation of *SCN1A* as a strategy for therapy of DS (Fig. 7).

## Summary

Approval of new drugs, including fenfluramine and cannabidiol in recent years, has led to a better control of seizures in DS. Especially fenfluramine seems to possess promising therapeutic properties, both through significant seizure reduction and intellectual enhancement. However, despite the increased number of therapeutic choices, DS is still therapy resistant in many cases, and besides reduction of seizures, there is an urgent need for new drugs with beneficial effects on other associated symptoms such as behavioral and cognitive deterioration. Drug repurposing of compounds such as lorcaserin and clemizole offers the advantage of a faster approval. Nevertheless, in the case of a disease whose origin can be traced back to a defect in a single gene, it is extremely important to develop new disease-modifying therapeutic concepts from the field of precision medicine. Furthermore, extended clinical evaluation and proof of effectiveness in placebo-controlled approaches of these new drugs in DS are still pending. In most cases, haploinsufficiency in DS is caused by truncating or missense mutations of *SCN1A*, leading to haploinsufficiency (Bechi and others 2012). In these cases, less stable protein is produced and an impairment of neuronal function by the mutated product is very unlikely. Thus, an enhancement of wild-type *SCN1A* expression could have great therapeutic potential. An antisense oligonucleotide directed against the alternative *SCN1A* transcript with inclusion of poison exon 20N, which leads to an upregulation of the wild-type transcript, is currently under investigation in a clinical phase 1 study (Carvill and others 2018; Han and others 2020; Helbig and Goldberg 2021; Voskobiynyk and others 2021). Also, a current approach using an antisense nucleotide directed against *SCN1A-dsAS*, which leads to an enhancement of *SCN1A* expression, shows some potential as a disease modifying concept for treatment of DS and awaits further clinical evaluation (Giagtzoglou 2021; Hsiao and others 2016). CRISPR/Cas technologies may modify specific DNA segments epigenetically and at the sequence level (Cong and others 2013; Dominguez and others 2016; Jinek and others 2012). An inactivated Cas9 enzyme fused to a transcriptional activator brought to the promoter region of *Scn1a* led to a better control of seizures in a haploinsufficient DS mouse model (Colasante and others 2020; Yamagata and others 2020). In recent years, new CRISPR-Cas technologies have revolutionized the field of gene therapy and will enable new concepts for clinical applications (Pickar-Oliver and Gersbach 2019). Nevertheless, in the rare case of a gain-of-function mutation in *SCN1A*, which was found in a recent study, upregulation of the transcript would most likely aggravate the clinical condition (Berecki and others 2019). Accordingly, before starting therapy, it is important to precisely characterize and classify the corresponding mutation.

With the development of specific drugs for DS, especially when aimed at the direct upregulation of intact *SCN1A*, the optimal time point of intervention is a matter of debate. Studies in mouse models of DS indicate that there could be a “point of no return” after which the transcriptome, proteome, and metabolome are altered in such a way that upregulation of *SCN1A* alone could no longer reverse these processes (Miljanovic and others 2021a, 2021b). Proteomic analysis of *Scn1a*-A1783V mice shows that in addition to ion channel dysfunction, the pathways of neurotransmitter signaling, synaptic plasticity, astrogliosis, and neoangiogenesis are affected by haploinsufficiency of *Scn1a* and that those modifications significantly increase over time (Miljanovic and others 2021a). In addition, new alterations occur progressively, such as an enrichment of proteins involved in glutamatergic signaling (Miljanovic and others 2021a). The analysis of the metabolome also shows that the energy production in the hippocampus of *Scn1a*-haploinsufficient mice is shifted toward catabolic processes with enhanced glycogenolysis and glycolysis and that over time, compensatory mechanisms, such as an increased GABA-to-glutamate ratio, develop (Miljanovic and others 2021b). These findings suggest that interventions aiming to enhance *SCN1A* wild-type expression in DS should be initiated as early as possible once the diagnosis is established. In order to assess the increase in Na_v_1.1 expression and function of these treatment approaches, a biomarker would be very helpful to predict and correlate the success of targeted therapy in patients with *SCN1A*-related DS.

While recent results are promising, much more work needs to be done to implement some of the reported approaches into clinical practice.
